# Factors governing the prevalence and richness of avian haemosporidian communities within and between temperate mountains

**DOI:** 10.1371/journal.pone.0184587

**Published:** 2017-09-07

**Authors:** Juan Carlos Illera, Guillermo López, Laura García-Padilla, Ángel Moreno

**Affiliations:** 1 Research Unit of Biodiversity, Oviedo University, Mieres, Asturias, Spain; 2 Instituto de Biología de la Conservación, Málaga, Spain; 3 Vice Council Environm, Serv. Environm. Impact, Las Palmas Gran Canaria, Canary Islands, Spain; Universidad de Granada, SPAIN

## Abstract

Mountains are well-suited systems to disentangle the factors driving distribution of parasites due to their heterogeneity of climatic and habitat conditions. However, the information about the relative importance of environmental factors governing the distribution of avian haemosporidians on temperate mountains is very limited. The main goal of the present study is to identify the factors determining prevalence and richness in avian haemosporidians (*Plasmodium*, *Haemoproteus* and *Leucocytozoon*) at the community level along elevational gradients on two mountain ranges located around the northern and southern limits of the Iberian Peninsula (Spain). We used samples from 68 avian species and 1,460 breeding individuals caught over widespread woodland and open habitats. Our findings confirmed the importance of climatic variables explaining prevalence and richness on Iberian mountains. However, landscape variables and other factors named host richness and migration behaviour explained more variation than climatic ones. *Plasmodium* genus preferred open and warm habitats. Water sources were also important for the southern but not for the northern mountain. *Haemoproteus* and *Leucocytozoon* showed affinities for woodland areas. Climatic conditions for *Haemoproteus* and *Leucocytozoon* were dependent on the mountain range suggesting some adaptation of avian haemosporidian and their invertebrate vectors to the climatic particularities of both mountain massifs. In contrast to *Plasmodium* and *Haemoproteus* genera, *Leucocytozoon* prevalence and richness values were significantly higher in the southern mountain range. Overall, our findings at the community level has enriched the relative weight and effect direction of environmental factors governing the distribution and prevalence of the avian haemosporidian community. Also, our results provide a caution message about the precision of predictive models on parasite distributions based on climatic variables, since such predictions could overestimate the effect of climate change scenarios on the transmission of the haemosporidians.

## Introduction

Understanding the ecological factors that determine the prevalence and richness of parasites in the wild has captivated the attention of ecologists and biogeographers for decades, as they provide the opportunity to predict pathogen proliferation in space and time [1, 2]. This matter is especially important in the dramatically growing human impact on Earth’s ecosystems such as climatic change, invasive species and land use [3, 4]. Such pressures may raise the proliferation of new diseases or increase the prevalence of others in the near future, which is an important cause for concern both human health and wildlife conservation [5, 6, 7].

One of the best studied parasite groups in wild animals are haemosporidians, especially those included in the genera *Plasmodium*, *Haemoproteus* and *Leucocytozoon*. These vector-borne parasites are protozoans infecting blood cells in many vertebrate species including humans [8]. Haemosporidians need intermediate and ectothermic hosts (i.e. arthropods) to complete their life cycle. This obligate relationship could promote specificities between parasite and invertebrate limiting the parasite transmission, which would only occur within the invertebrate host distribution [9, 10]. Our knowledge of host-parasite relationships in non-human haemosporidian pathogens has noticeably increased during the last decade, largely due to the routine use of molecular markers [11, 12]. Within vertebrate hosts, avian species have emerged by far as the group with most attention on such relationships [13, 14, 15]. Such an unprecedented data throughput has provided new understandings on host-parasite relationships related to biogeographical, ecological and evolutionary topics [16, 17, 18, 19].

Climatic features have been identified as important factors explaining distribution of both haemosporidians and their vectors, since they require availability of water and specific temperature conditions to complete their development and transmission processes [20, 21, 22]. However, recent research has highlighted that other predictors aside from climate, such as landscape features and anthropogenic factors, also influence prevalence and richness of avian blood parasites [23]. In addition, the picture is far from homogenous because these results have revealed that haemosporidians from different genera can show different responses to similar environmental conditions [24, 25]. Most of these studies were evaluated on single species or local communities inhabiting particular ecological niches such as forests and semi-arid habitats [1, 24, 26], but see [25]. Undoubtedly, these results have provided insights in to how environmental factors determine prevalence and richness of parasites. However, it seems necessary to perform analyses of prevalence and diversity at the community level, incorporating information from heterogeneous avian communities and habitats together in order to deepen our conceptual framework about the parasite distributions at larger spatial scales with higher ecological diversity. These approaches are especially needed since we know that many haemosporidian lineages are less specific than have been believed [25, 27]. Therefore, conclusions about the environmental factors determining prevalence and richness of parasites obtained with single avian hosts or local communities can probably not be applied to heterogeneous avian communities.

Mountains show a great heterogeneity of climatic and habitat conditions due to their elevational dimension, which determines significant changes in environmental conditions over short geographic distances and promotes variation in life histories and morphological traits [28, 29, 30]. Such systems are well-suited to determine the factors driving distribution and prevalence of parasites [31, 32, 33]. Elevational gradients also provide an exceptional framework to monitor and quantify long-term changes in avian communities with time [34, 35], which could be influenced by parasites [36]. Despite improvements in our knowledge of patterns of haemosporidian distributions along tropical and oceanic mountains [31, 32, 25], the information about the relative importance of environmental factors governing the prevalence and richness of haemosporidians on temperate mountains is very limited. However, this information would allow us to predict future parasite distribution (including potential impact on their hosts) under singular climatic change scenarios [2, 37].

The main goal of the present study is to identify the factors determining prevalence and richness in avian haemosporidians at the community level along elevational gradients of two mountain ranges. The mountains are located in the northern (Cantabrian Mountains) and southern (Sierra Nevada) limits of the Iberian Peninsula (Spain). Both mountain ranges are characterised to contain most environmental features (including the oceanic and Mediterranean climates) representative of the Iberian Peninsula [38]. In addition, the geographical position of both mountains (≈ 700 km away each other), and the highest altitudes above 2,600 m provide an ideal scenario to compare the elevational effect dependent on latitude. To identify the appropriate variables explaining haemosporidian distributions we used a wide set of 51 environmental predictors, which describe climatic (temperature and rainfall), landscape (including topographical and anthropogenic variables), and geographical characteristics. We will perform partial least squares regressions to analyse the data set, which is a well-suited statistical method to interpret the effect of many environmental predictors on organism distributions [24]. Based on previous findings we expect to find a strong effect of climatic variables, and a lesser extent of landscape characteristics, explaining prevalence and richness of avian haemosporidians [24, 31, 39]. Climatic features such as temperature and water availability are important factors explaining haemosporidian infection since they play a significant role are key during vector larval development and adult survival [22, 40]. Moreover, we expect to find a distinct response for each haemosporidian genera. Thus, we predict to find the highest prevalence values for *Plasmodium* parasites at lower but warmest altitudes. This prediction is related with the constant warm temperatures that this genus needs to complete its life cycle [20, 25]. However, based on previous studies we predict that we will find the opposite trend for *Leucocytozoon* and *Haemoproteus*, that is, they would achieve the highest prevalence values at higher and cooler altitudes, which could be related with the ecological requirements of their dipteran vectors [14, 23]. In general, we expect to find higher lineage diversity for *Leucocytozoon* and *Haemoproteus*, but wider distributions for *Plasmodium* since this genus appears to be more cosmopolitan than the other two genera [23]. However, we predict to find similar results on both prevalence and richness in the haemosporidian parasites [24]. Overall, these results will improve our understanding of the environmental factors determining the distribution of avian blood parasites infecting a multi-host community in temperate mountains. Importantly, such information will provide a solid framework of biotic and abiotic variables for developing robust predictive models of haemosporidian distributions, which will help to predict future changes in the parasite distribution in light of the different climatic and human-induced scenarios expected to occur.

## Material and methods

### Ethics statement

This project was approved and funded by the Spanish Administration of National Parks, Spanish Minister of Agriculture, Food and Environment (Ref.: 375/2011). The funds obtained for performing this project were applied last 2011, however, the ethical committee in Asturias was created last 2014. Therefore, the committee started to evaluate projects after the ending of this study. The Regional Governments of Andalusia, Asturias and Castilla y León, and the National Parks of Sierra Nevada and Picos de Europa gave permission to conduct fieldwork and blood sampling. All animal sampling work was conducted according Spanish guidelines.

#### Study area and sampling

**Fig 1:**
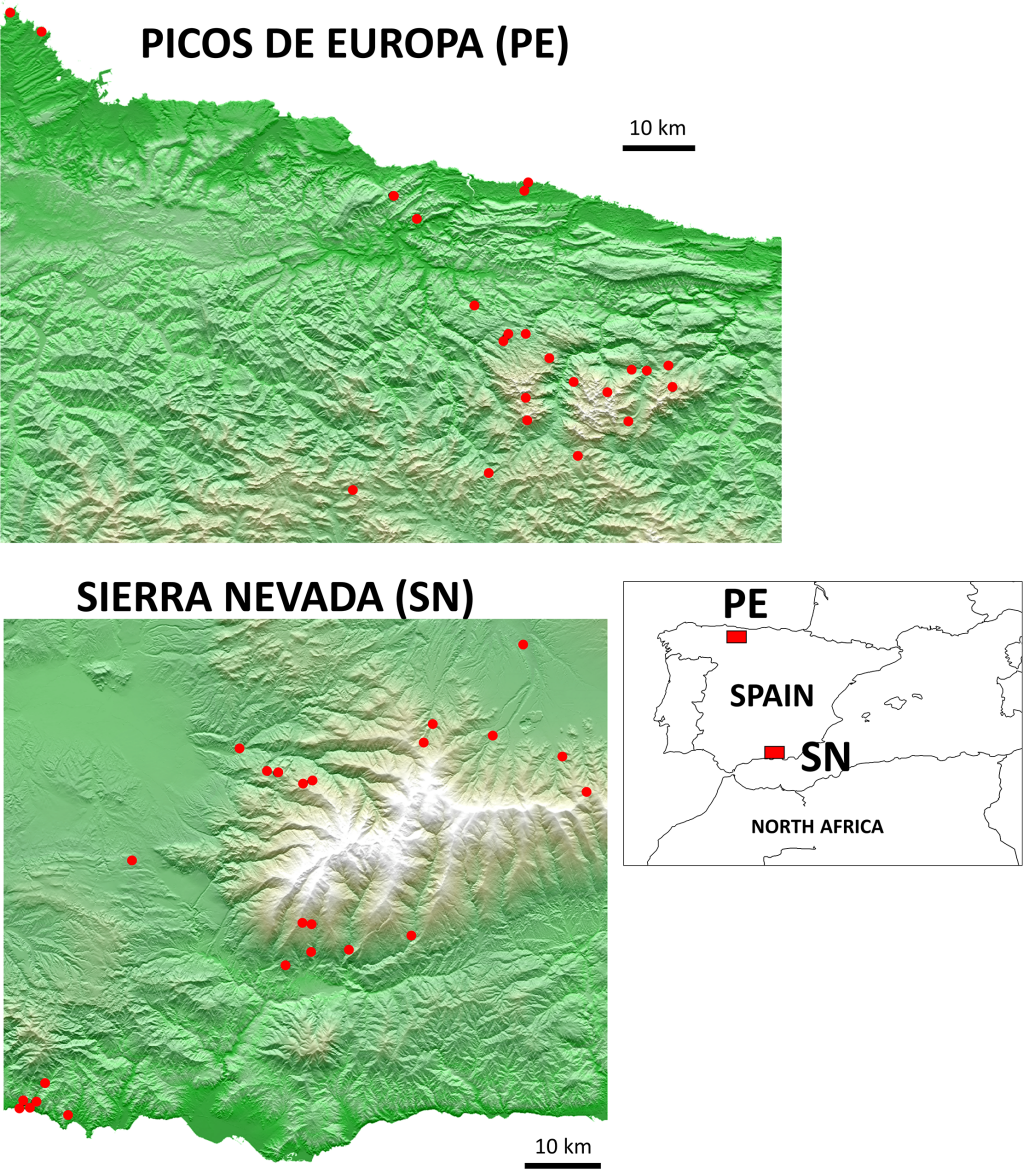
Geographical position of the mountain ranges studied. Red dots show the localities sampled. Picos de Europa and Sierra Nevada maps were depicted from free information obtained from the Spanish Geographic National Institute (http://centrodedescargas.cnig.es/CentroDescargas/index.jsp#) and using the program Geographic Resources Analysis Support System (GRASS), version 6.

Our study area included two mountain ranges included within the Spanish National Park network: Sierra Nevada and Picos de Europa, located in the south-east and the north-west of the Iberian Peninsula, respectively (Fig 1).

Sierra Nevada is a north east-trending Palaeozoic metamorphic massif that includes the highest altitude in the Iberian Peninsula (3,478 m a.s.l.; 37°03’12”N, 3°18’41”W). Climate conditions are typically Mediterranean but with specific characteristics of a semi-arid mid-latitude mountain region, with annual mean temperatures of ~15°C and annual precipitation ranging from 800 mm/yr in the highlands to < 400 mm/yr in the lowlands [41]. Predominant forests are composed of pines (*Pinus spp*.), holm oaks (*Quercus ilex*), and other semi-deciduous oaks (*Quercus spp*.), whereas open areas include different Mediterranean scrubland and pastureland communities [42]. Picos de Europa is a west east-trending Mesozoic karstic massif reaching its highest altitude at 2,648 m a.s.l. (43° 11’ 52”N, 4° 51’ 09”). Climate is typically Eurosiberian, with mean annual temperatures of ~13°C and annual precipitation exceeding 1,500 mm/yr in the northern slopes [43]. Woodland areas are dominated by semi-deciduous oaks (*Quercus spp*.), birches (*Betula alba*), and beeches (*Fagus sylvatica*), whereas elements from Ericaceae and Fabaceae families dominate the open areas [44]. In order to capture as much variability as possible along the bioclimatic gradient of the sampling area, we defined four-tier altitudinal ranges: (1) from 28 m a.s.l. to 545 m; (2) from 760 m to 1170 m; (3) from 1250 m to 1620 m; and (4) from 1835 m to 2090 m (Fig 1). We sampled six localities (three in forest habitats and three in open habitats) on each defined range (except in the highest range of Picos de Europa where only three localities were sampled because of the lack of forests above 1600 m). In addition, we sampled an extra locality from the 1250–1620 m range in Picos de Europa. In total we sampled 46 localities (24 in Sierra Nevada and 22 in Picos de Europa) (S1 Table, Fig 1). The sampling period coincided with the avian breeding season: April-June in Sierra Nevada and May-July in Picos de Europa of 2012 and 2013.

We captured birds using mist and clap nets. We spent one ringing day per locality and year, except in Picos de Europa where we increased the sampling effort in the alpine habitats (i.e. levels 3 and 4) up to five days per locality due to the difficulty of reaching these localities. Most of individuals (93%) were adult birds, and juveniles were, at least, two months old. We ringed all birds with a unique numbered aluminium ring provided from the Spanish authorities. Birds were also examined with extreme care for evidence of poxvirus lesions [45]. We collected a small blood sample (20–40 μl) by brachial or jugular venipuncture using sterile insulin syringes. Blood was preserved in around 800–1000 μl of absolute ethanol in screw-cap tubes, and stored at room temperature. All birds were released in the same places where they were trapped.

#### Molecular procedures

DNA was routinely extracted using an ammonium acetate technique [46], except in a few samples where a commercial DNA extraction kit (Qiagen’s DNeasy kit) was used. Despite some bird species were dimorphic we sexed all individuals with the primers (P2 and P8) and PCR conditions specified in Griffiths et al. [47]. This is a common procedure when a negative amplification is also a valid result, and it is necessary to ensure that we used amplifiable DNA [48, 49].

To detect the haemosporidian parasites (i.e. *Plasmodium*, *Haemoproteus*, and *Leucocytozoon*) we used a nested PCR method described in Hellgren et al. [12] that amplifies a fragment of 478 (for *Leucocytozoon*) or 480 (for *Plasmodium* and *Haemoproteus*) base pairs (bp) of the mitochondrial cytochrome b gene. For the first PCR we used the primers HaemNFI and HaemNR3. For the second reaction, we used the primers HaemF and HaemR2 to amplify *Plasmodium*/*Haemoproteus*, and HaemFL and HaemR2L to amplify *Leucocytozoon* haemosporidians [12]. For PCR reactions, we followed the conditions and reagents described in Illera et al. [49]. We used two positive and negative (water) controls per PCR reaction. All positive amplifications detected on the agarose gel after electrophoresis, and corresponding with the expected size of the cytochrome b gene, were sequenced. Sequences were edited and aligned by eye against homologous sequences [48, 49], using BIOEDIT v. 7.2.5. [50] and Chromas Lite version 2.1.1. (http://technelysium.com.au/?page_id=13). All sequences were BLAST in the NCBI gene bank database to determine the haemosporidian genus. We replicated twice the screening to check repeatability and ensure the accuracy of results. All sequences found to show double peaks were amplified and sequenced again, and where results were concordant they were considered as double infections. Here we define double infection as birds infected with more than one haemosporidian lineage, and which cannot be unequivocally identified with the primers used. We found 15 cases compatible with double infections, and we inferred the mitochondrial haplotypes using the program DnaSP version 5.10.01 [51] and the algorithm PHASE [52, 53] there implemented [32].

#### Predictor variables

Most of the sampled species are territorial during the breeding period with limited movements out of their home ranges. Thus, we defined an area of 1 km^2^ around each sampled locality to roughly characterise the biotic and abiotic conditions. In total, we used a set of 51 environmental variables (Tables 1–3). We obtained the bioclimatic variables (temperature and rainfall) from BIOCLIM (www.worldclim.org/bioclim), which provided average data for the years 1970–2000. Here we have to assume that any climatic change occurred since this period had to be of a similar intensity for all localities studied. The topographic features from the Spanish Geographic Institute (https://www.ign.es/ign/main/index.do?locale=en), and land use variables from CORINE 2000 Land cover (https://www.eea.europa.eu/data-and-maps/data/clc-2000-2006-changes-5). In addition, we used latitude and longitude to control for spatial structure of data. Finally, we used three predictors to evaluate the migratory behaviour (number of migratory individuals divided between the number of sedentary individuals per locality), sex ratio (number of males divided between the number of females per locality), and host-richness (number of avian species sampled per locality), which could be considered a rough proxy of the bird community present on each locality. Geographic Information System (GIS) analyses were performed with the program Geographic Resources Analysis Support System (GRASS), version 6.4 (www.grass.osgeo.org).

**Table 1:** Partial least squares regression results for *Plasmodium* prevalence and richness.

Predictors	*Plasmodium*							
	Sierra Nev. & Picos Eur.		Sierra Nevada		Picos de Europa			
	Prevalence	Richness	Prevalence	Richness	Prevalence		Richness	
	1 Component	1 Component	1 Component	1 Component	1st Component^§^	2nd Component^§^	1st Component^§^	2nd Component^§^
**Confounding variable**								
Sex ratio			**0.190**	**0.292**				
**Geographical features**								
Longitude (X)					**-0.187**		**-0.181**	
Latitude (Y)			**-0.150**		**0.208**		**0.223**	
X*Y					**-0.180**		**-0.171**	
X^2^					**-0.187**		**-0.181**	
Y^2^			**-0.200**	**-0.178**	**0.188**	**0.156**	**0.197**	
X^2^*Y					**-0.183**		**-0.178**	
X*Y^2^	**-0.280**	**-0.249**			**-0.179**		**-0.175**	
X^3^					**-0.186**		**-0.179**	
Y^3^			**-0.263**	**-0.228**	**0.207**		**0.217**	
**Climatic**								
***Temperature***								
Annual mean	**0.249**	**0.221**			**0.146**	**-0.147**		
Mean diurnal range					**-0.182**		**-0.196**	
Isothermality			**0.171**	**0.203**	**0.184**		**0.175**	
Temperature seasonality					**-0.200**		**-0.207**	
Max. temperature warmest month	**0.159**	**0.156**	**0.183**	**0.168**		**-0.209**		**-0.186**
Min. temperature coldest month			**-0.180**	**-0.180**				
Annual Range					**-0.195**		**-0.205**	
Mean temperature wettest quarter	**0.205**	**0.158**				**-0.151**		
Mean temperature driest quarter	**0.217**	**0.200**			**0.144**	**-0.158**		
Mean temperature warmest quarter	**0.220**	**0.203**			**0.145**	**-0.156**		
Mean temperature coldest quarter	**0.238**	**0.216**	**0.154**					
***Precipitation***								
Annual precipitation	**-0.148**					**0.231**		**0.204**
Precipitation of wettest month						**0.303**		**0.263**
Precipitation of driest month						**0.209**		**0.180**
Precipitation seasonality	**0.159**	**0.159**	**0.142**		**0.164**		**0.158**	
Precipitation of wettest quarter	**-0.152**	**-0.145**				**0.232**		**0.210**
Precipitation of driest quarter						**0.196**		**0.172**
Precipitation of warmest quarter	**-0.141**	**-0.148**	**-0.149**			**0.231**		**0.208**
Precipitation of coldest quarter	**-0.172**	**-0.148**				**0.207**		**0.186**
**Landscape features**								
Altitude	**-0.231**	**-0.194**			**-0.168**		**-0.174**	
North-South Orientation								**0.188**
East-West Orientation		**-0.144**				**-0.165**		**-0.176**
Slope					**-0.163**		**-0.189**	
Distance to permanent rivers						**0.155**		
Distance to temporary rivers								**0.178**
Distance to water channels								
Distance to permanent lakes			**-0.284**	-**0.252**		**0.171**		
Distance to temporary lakes							**0.151**	
Distance to reservoir waters	**0.182**				**0.197**		**0.189**	
Distance to drinking fountains			**-0.147**		**0.171**		**0.145**	
Distance to spring water	**0.251**	**0.180**			**0.171**		**0.145**	
Distance to water tanks			**-0.181**	**-0.196**	**0.159**			
Distance to water wells			**-0.193**	**-0.198**	**0.167**	**0.171**	**0.156**	
Distance to ponds	**-0.194**	**-0.233**					**-0.178**	**-0.218**
Distance to urban areas	**-0.146**	**-0.242**		**-0.213**				
Percentage of agricultural areas	**0.185**	**0.212**	**0.205**	**0.202**				
Percentage of forests	**-0.209**	**-0.330**	**-0.319**	**-0.352**				**-0.234**
Percentage of soil				**0.207**				
Percentage of shrubs						**0.188**		**0.180**
**Behaviour**								
Migration						**0.207**		**0.423**
**Number of potential hosts**								
Host richness	**-0.161**	**-0.185**	**-0.191**	**-0.178**		**-0.247**		**-0.190**

Weights of predictors (independent variables) are related to prevalence and richness (dependent variables) for *Plasmodium* on each mountain range and for the pooled analysis (i.e. Sierra Nevada plus Picos de Europa). Only values found to contribute significantly to the component obtained are shown (see S2–S4 Tables for the full list of values per variable).

^**§**^: Significant component.

**Table 2:** Partial least squares regression results for *Haemoproteus* prevalence and richness.

Predictors	*Haemoproteus*							
	Sierra Nev. & Picos Eur.		Sierra Nevada				Picos de Europa	
	Prevalence	Richness	Prevalence		Richness		Prevalence	Richness
	1 Component	1 Component	1st Component^§^	2nd Component	1st Component^§^	2nd Component	1 Component	1 Component
**Confounding variable**								
Sex ratio						**-0.204**		
**Geographical features**								
Longitude (X)	**0.182**	**0.173**	**0.213**		**0.216**			
Latitude (Y)			**0.192**		**0.176**			
X*Y	**0.217**	**0.215**	**0.196**		**0.203**			
X^2^	**0.185**	**0.175**	**0.215**		**0.218**			
Y^2^			**0.193**		**0.156**			
X^2^*Y	**0.204**	**0.196**	**0.211**		**0.211**			
X*Y^2^	**0.242**	**0.245**	**0.225**		**0.221**			
X^3^	**0.180**	**0.172**	**0.208**		**0.215**			
Y^3^								
**Climatic**								
***Temperature***								
Annual mean			**-0.179**		**-0.177**			
Mean diurnal range	**0.199**	**0.164**		**-0.168**		**-0.207**		
Isothermality				**0.207**				
Temperature seasonality	**0.186**	**0.159**	**0.151**			**-0.163**		
Max. temperature warmest month			**-0.161716**		**-0.176**		**0.203**	**0.186**
Min. temperature coldest month	**-0.218**	**-0.239**					**-0.248**	**-0.294**
Annual Range	**0.192**	**0.163**		**-0.152**		**-0.184**		
Mean temperature wettest quarter			**-0.153**		**-0.143**		**0.145**	
Mean temperature driest quarter			**-0.179**		**-0.177**			
Mean temperature warmest quarter			**-0.178**		**-0.176**			
Mean temperature coldest quarter			**-0.155**		**-0.154**			
***Precipitation***								
Annual precipitation			**0.174**		**0.166**		**-0.212**	**-0.197**
Precipitation of wettest month			**0.165**		**0.156**		**-0.286**	**-0.287**
Precipitation of driest month			**0.187**		**0.172**		**-0.166**	**-0.150**
Precipitation seasonality			**-0.212**		**-0.206**			
Precipitation of wettest quarter			**0.163**		**0.154**		**-0.229**	**-0.214**
Precipitation of driest quarter			**0.196**		**0.187**		**-0.163**	
Precipitation of warmest quarter			**0.201**		**0.206**		**-0.210**	**-0.195**
Precipitation of coldest quarter			**0.150**		**0.142**		**-0.196**	**-0.181**
**Landscape features**								
Altitude	**0.183**	**0.162**	**0.174**		**0.162**			
North-South Orientation				**0.172**				
East-West Orientation								
Slope						**0.141**		
Distance to permanent rivers	**-0.243**	**-0.165**		**0.268**		**0.246**	**-0.338**	**-0.249**
Distance to temporary rivers		**-0.181**				**-0.177**	**-0.208**	**-0.214**
Distance to water channels				**0.166821**				
Distance to permanent lakes								
Distance to temporary lakes				**0.201386**		**0.212**		
Distance to reservoir waters		**-0.166**						
Distance to drinking fountains								
Distance to spring water						**0.225**		
Distance to water tanks				**0.235**		**0.214**		
Distance to water wells				**0.398**		**0.328**		
Distance to ponds	**0.166**						**0.142**	**0.200**
Distance to urban areas								
Percentage of agricultural areas								
Percentage of forests	**0.314**	**0.278**			**0.158**		**0.238**	**0.189**
Percentage of soil	**-0.213**	**-0.181**		**-0.240**		**-0.148**	**-0.150**	**-0.159**
Percentage of shrubs				**0.229**				
**Behaviour**								
Migration	**-0.253**	**-0.236**		**0.295**		**-0.335**	**-0.269**	**-0.206**
**Number of potential hosts**								
Host richness	**0.397**	**0.485**	**-0.048**	**-0.281**	**0.290**	**0.415**	**0.191**	**0.303**

Weights of predictors (independent variables) are related to prevalence and richness (dependent variables) for *Haemoproteus* on each mountain range and for the pooled analysis (i.e. Sierra Nevada plus Picos de Europa). Only values found to contribute significantly to the component obtained are shown (see S2–S4 Tables for the full list of values per variable).

^**§**^: Significant component.

**Table 3:** Partial least squares regression results for *Leucocytozoon* prevalence and richness.

Predictors	*Leucocytozoon*										
	Sierra Nevada & Picos de Europa				Sierra Nevada		Picos de Europa				
	Prevalence		Richness		Prevalence	Richness	Prevalence			Richness	
	1st Comp. ^§^	2nd Comp.	1st Comp. ^§^	2nd Comp.	1 Comp.	1 Comp.	1st Comp. ^§^	2nd Comp. ^§^	3rd Comp.	1st Comp. ^§^	2nd Comp. ^§^
**Confounding variable**											
Sex ratio						**-0.157**					
**Geographical features**											
Longitude (X)	**0.173**		**0.181**						**-0.182**		**0.157**
Latitude (Y)	**-0.193**		**-0.194**		**-0.176**			**-0.212**			**-0.226**
X*Y			**0.147**						**-0.201**		
X^2^	**0.173**		**0.182**						**-0.188**		**0.157**
Y^2^	**-0.197**		**-0.196**		**-0.302**			**-0.276**			**-0.235**
X^2^*Y	**0.162**		**0.174**						**-0.187**		**0.154**
X*Y^2^									**-0.203**		
X^3^	**0.174**		**0.182**			**0.144**			**-0.174**		**0.157**
Y^3^	**-0.191**		**-0.193**						**0.270**		**-0.160**
**Climatic**											
***Temperature***											
Annual mean	**0.170**		**0.171**				**0.186**			**0.194**	
Mean diurnal range								**0.219**			**0.196**
Isothermality		**0.219**		**0.217**	**0.188**	**0.208**	**0.178**		**0.205**	**0.156**	
Temperature seasonality								**0.182**			**0.181**
Max. temperature warmest month	**0.203**		**0.209**			**-0.234**	**0.223**			**0.232**	
Min. temperature coldest month		**-0.148**			**-0.167**				**0.200**	**-0.147**	**-0.178**
Annual Range								**0.201**			**0.191**
Mean temperature wettest quarter							**0.185**			**0.194**	
Mean temperature driest quarter	**0.193**		**0.195**			**-0.148**	**0.186**			**0.195**	
Mean temperature warmest quarter	**0.192**		**0.196**			**-0.148**	**0.183**			**0.194**	
Mean temperature coldest quarter							**0.155**			**0.166**	
***Precipitation***											
Annual precipitation	**-0.178**		**-0.170**				**-0.222**			**-0.236**	
Precipitation of wettest month	**-0.187**		**-0.176**				**-0.227**		**0.230**	**-0.253**	
Precipitation of driest month	**-0.177**		**-0.169**				**-0.197**			**-0.213**	
Precipitation seasonality	**0.196**		**0.194**				**0.182**			**0.183**	
Precipitation of wettest quarter	**-0.182**		**-0.173**				**-0.222**		**0.151**	**-0.238**	
Precipitation of driest quarter	**-0.176**		**-0.169**				**-0.197**			**-0.212**	
Precipitation of warmest quarter	**-0.171**		**-0.164**	**0.152**		**0.146**	**-0.212**		**0.145**	**-0.231**	
Precipitation of coldest quarter	**-0.166**		**-0.162**				**-0.222**			**-0.231**	
**Landscape features**											
Altitude								**0.222**			**0.176**
North-South Orientation		**-0.142**									
East-West Orientation											
Slope	**0.148**				**0.233**	**0.174**		**0.279**			**0.222**
Distance to permanent rivers				**-0.146**				**-0.149**			**-0.150**
Distance to temporary rivers	**-0.142**		**-0.161**	**-0.163**	**-0.174**	**-0.152**				**-0.176**	**-0.199**
Distance to water channels	**-0.149**										
Distance to permanent lakes					**-0.205**				**0.217**		
Distance to temporary lakes									**-0.165**		
Distance to reservoir waters		**0.152**							**0.268**		
Distance to drinking fountains								**-0.185**			**-0.188**
Distance to spring water					**0.263**			**-0.185**			**-0.188**
Distance to water tanks											
Distance to water wells								**-0.155**			**-0.197**
Distance to ponds					**-0.214**						**0.134**
Distance to urban areas											
Percentage of agricultural areas		**0.163**			**0.279**						
Percentage of forests		**0.348**		**0.375**		**0.186**	**0.253**	**0.360**	**0.238**	**0.194**	**0.275**
Percentage of soil	**-0.175**		**-0.174**				**-0.239**	**-0.177**		**-0.175**	
Percentage of shrubs		**-0.351**		**-0.289**	**-0.381**	**-0.331**					
**Behaviour**											
Migration	**-0.191**	**-0.396**	**-0.183**	**-0.358**	**-0.300**	**-0.309**	**-0.283**	**-0.200**	**-0.232**	**-0.249**	**-0.145**
**Number of potential hosts**											
Host richness	**0.162**	**0.469**	**0.196**	**0.562**	**0.331**	**0.465**	**0.203**	**0.212**		**0.234**	**0.255**

Weights of predictors (independent variables) are related to prevalence and richness (dependent variables) for *Leucocytozoon* on each mountain range and for the pooled analysis (i.e. Sierra Nevada plus Picos de Europa). Only values found to contribute significantly to the component obtained are shown (see S2–S4 Tables for the full list of values per variable).

^**§**^: Significant component.

#### Statistical procedures

We performed partial least squares regression (PLS) to predict the prevalence and richness of each haemosporidian genus using our set of 51 climatic, landscape and anthropic predictors. Our response variables were prevalence and richness per haemosporidian genus and locality. This statistical approach is recommendable when the number of independent variables is large in relation to the number of observations, but also when predictors are highly correlated or even collinear [54, 55]. This analysis is particularly suitable when there is no reason to limit the number of measured factors because, for instance, we do not have a good understanding of how important these factors are to the response variables, a common circumstance in ecological studies [24, 56]. We computed PLS analyses using the standard nonlinear iterative partial least squares (NIPALS) algorithm implemented in the program STATISTICA 8.0 (StatSoft. Inc. 1984–2007). We used a maximum number of iterations of 300, and a convergence criterion value of 10^−4^. To evaluate the model performance and the number of components we used the sevenfold cross-validation method, which repeatedly leaves out one-seventh of the samples to validate how well the model describes the data. Only components explaining more than 10% of variance were selected. To determine the contribution of each predictor, we compared the square predictor weight in relation to the inverse of the number of independent variables (k). Because the sum of the square predictor weights is 100% of the explained variance, all variables with a square weight higher than 1/k were considered to contribute significantly to the component obtained [24]. We considered richness as the number of unique sequences found (i.e. sequences with at least one bp difference) per locality, and prevalence as the proportion of individuals infected per locality. We performed a full PLS including the 46 sampling localities (hereafter pooled analysis), and two PLS analysing the localities from Sierra Nevada (n = 24) and Picos de Europa (n = 22) independently (hereafter separated analysis). In addition, we performed univariate t-tests to compare prevalence and richness between Sierra Nevada and Picos de Europa. All variables were arcsin (proportion) or log-transformed (continuous data).

## Results

**Fig 2:**
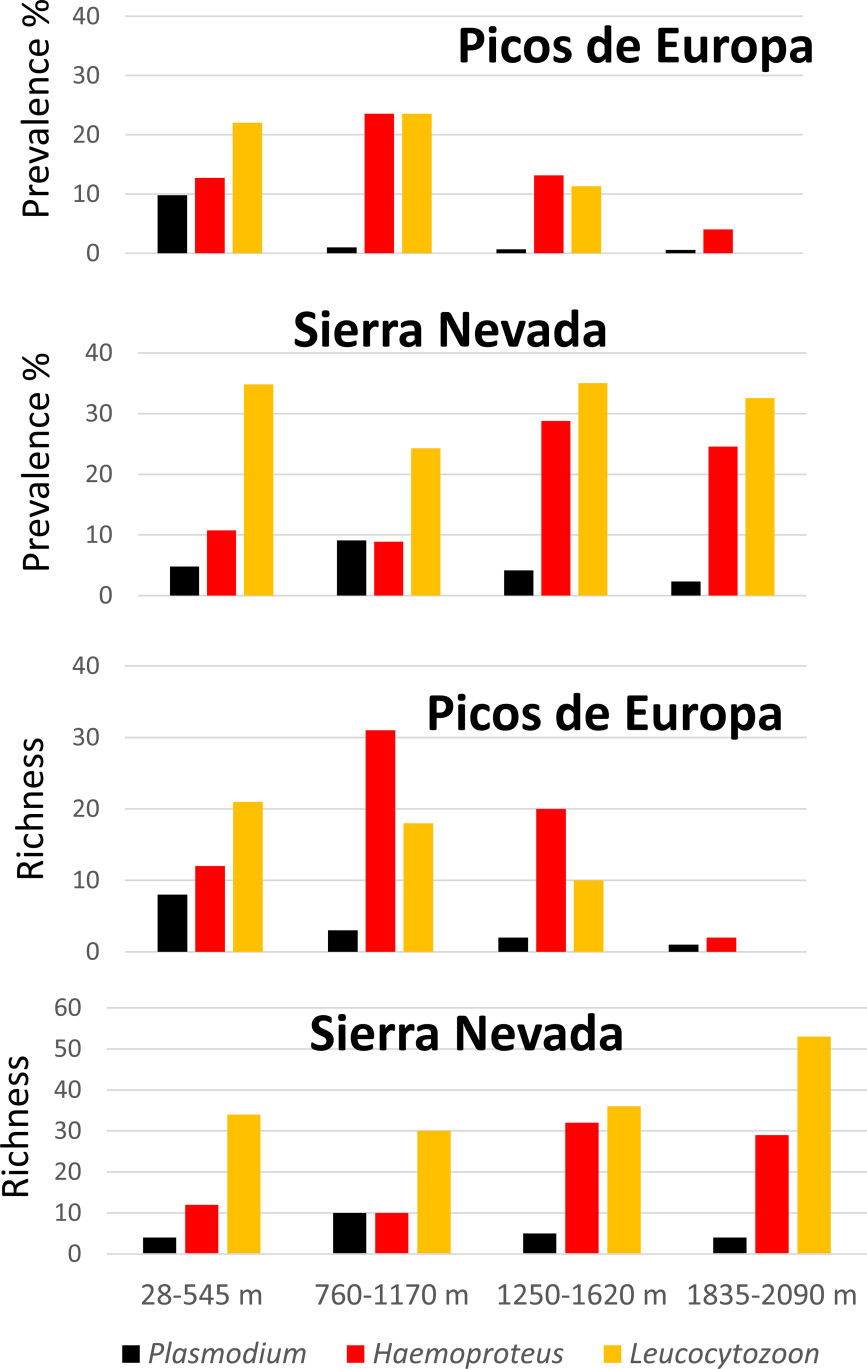
Prevalence and richness of *Plasmodium*, *Haemoproteus* and *Leucocytozoon* along four altitudinal ranges in Sierra Nevada and Picos de Europa.

A total of 1,460 individuals (645 and 802, for Sierra Nevada and Picos de Europa, respectively) from 68 species were caught (53 and 42 species for Sierra Nevada and Picos de Europa, respectively), and screened. Individuals from non-breeding species trapped in the localities sampled were excluded. Most of species (82%) and individuals (82%) sampled were sedentary. We did not find any case of lesions compatible with poxvirus infection. Our PCR results were consistent between repeated PCRs (95% of repeatability). We did not find significant differences in the haemosporidian prevalence or richness between the two years studied either for Sierra Nevada (t > -0.9, df = 46, p > 0.17) nor for Picos de Europa (t < 2.1, df = 42, p > 0.09), except for *Plasmodium* richness in Picos de Europa (t = 2.03, df = 42, p = 0.02). Hence, we analysed the results of both years together to maximise the statistical power of tests. The mean number of birds captured per locality was 26.62 ± 2.02 in Sierra Nevada and 36.41 ± 4.31 in Picos de Europa. We found low-moderate prevalence values for *Plasmodium* (4.65%, 2.24%), *Haemoproteus* (19.22%, 10.47%), and *Leucocytozoon* (32.56%, 7.98%), for Sierra Nevada and Picos de Europa, respectively (Fig 2).

We did not find significant differences of prevalence and richness between Sierra Nevada and Picos de Europa for *Plasmodium* and *Haemoproteus* (t > 0.16, df = 41, p > 0.15). However, Sierra Nevada showed significantly higher prevalence (t = -3.34, df = 41, p = 0.002) and richness (t = 4.05, df = 41, p < 0.001) of *Leucocytozoon* protozoans. In total, we found 15 cases with double infections, 12 in Sierra Nevada and three in Picos de Europa. All except one bird corresponded with double infections produced by the same haemosporidian genus: 7 *Leucocytozoon*, 6 *Haemoproteus*, and 1 *Plasmodium*. The exception was a cirl bunting (*Emberiza cirlus*) who showed a double infection produced by *Plasmodium* and *Haemoproteus* lineages.

We recorded a total of 126 haemosporidian haplotypes (13 *Plasmodium*, 41 *Haemoproteus*, and 72 *Leucocytozoon*) for Sierra Nevada, and 75 (7 *Plasmodium*, 36 *Haemoproteus*, and 32 *Leucocytozoon*) for Picos de Europa. New haplotypes have been deposit in the National Centre for Biotechnology Information (NCBI) gene bank database with the following accession numbers: MF625089—MF625253.

**Fig 3:**
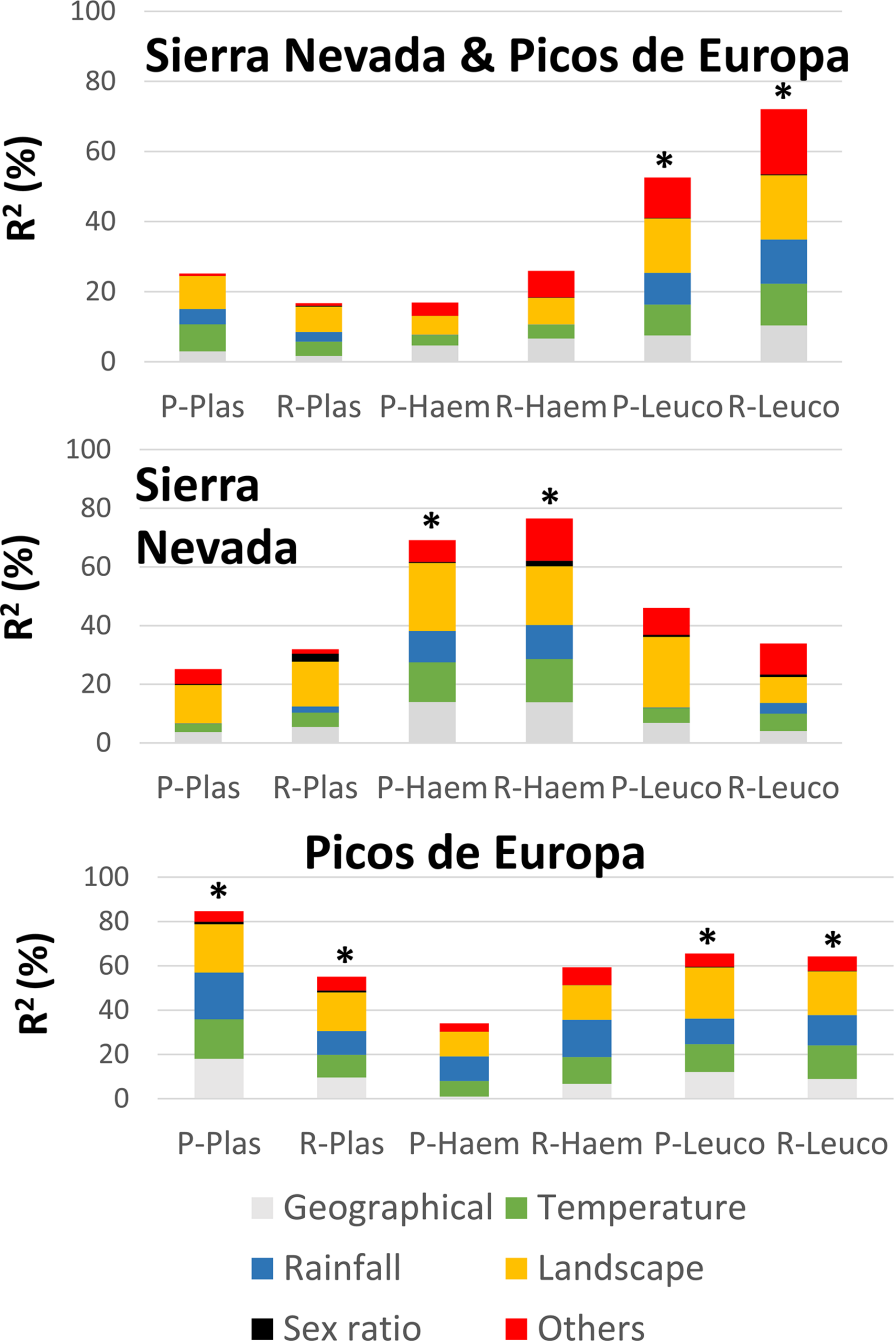
Percentage of variance explained (R^2^) after partial least squares regression analyses performed for prevalence and richness of *Plasmodium*, *Haemoproteus* and *Leucocytozoon*. Relative contribution of five groups of predictors are depicted with different colours. Results are shown by type of analyses: pooled (Sierra Nevada and Picos de Europa) and separated analyses for each mountain range. P-Plas: *Plasmodium* prevalence. R-Plas: *Plasmodium* richness. P-Haem: *Haemoproteus* prevalence. R-Haem: *Haemoproteus* richness. P-Leuco: *Leucocytozoon* prevalence. R-Leuco: *Leucocytozoon* richness. *: Significant component.

PLS analyses provided between one and three components (Tables 1–3). Prevalence and richness provided similar trends both in number of components selected and direction of effect size (Tables 1–3, Fig 3). The percentage of explained variance was higher on the separated analyses than in the pooled analysis, except for *Leucocytozoon* (Fig 3).

*Plasmodium* prevalence and richness was inversely associated with altitude and forest coverage, but positively associated with agricultural areas in the pooled and separated PLS analyses. This result suggests that avian *Plasmodium* are favoured in low altitude and open habitats across the two mountain ranges. The pooled analyses showed that *Plasmodium* prevalence and richness increased with temperature and, in general, was negatively related to rainfall variables. Anthropogenic factors, such as distance to urban areas and ponds, showed a negative association with prevalence and richness, that is, prevalence and richness values were higher in the proximity of these two predictors. In addition, host richness showed a negative relationship with *Plasmodium* prevalence and richness, which means that both dependent variables decreased with the number of avian species sampled (Table 1). Separated analyses found a negative effect of latitude over *Plasmodium* prevalence in Sierra Nevada, but a positive effect over *Plasmodium* prevalence and richness in Picos de Europa. This result is congruent in both mountain ranges with a pattern of *Plasmodium* prevalence and richness decreasing from the coastal to the inland, that is, southern-northern direction for Sierra Nevada, and northern-southern for Picos de Europa. Sex ratio showed a positive effect over *Plasmodium* prevalence and richness in Sierra Nevada, suggesting that this genus is thriving in sites with the greater presence of males (Table 1).

The pooled analysis showed that *Haemoproteus* prevalence and richness were positively related to longitude, altitude, woodland coverage and host richness, and negatively related with the sedentary behaviour (Table 2). There was not a significant effect between *Haemoproteus* and rainfall variables. Separated analyses showed a positive association between *Haemoproteus* prevalence and richness, and latitude and longitude in Sierra Nevada, but we did not find such a relationship in Picos de Europa. Rainfall was positively related to *Haemoproteus* prevalence and richness in Sierra Nevada, but negatively related to *Haemoproteus* prevalence and richness in Picos de Europa. Temperature was negatively related to *Haemoproteus* prevalence and richness in Sierra Nevada (Table 2).

*Leucocytozoon* prevalence and richness showed a positive association with temperature and a negative association with rainfall in the pooled analysis. Woodland coverage was a positive predictor of *Leucocytozoon* prevalence and richness, but soil and shrub coverage showed a negative association. Hence, our results suggest that *Leucocytozoon* is favoured in warm and dry woodlands. *Leucocytozoon* showed a significant positive relationship with host richness, but a negative relationship with host migratory behaviour. This result suggests that avian *Leucocytozoon* prevalence and richness is favoured in areas with high host diversity where sedentary species are predominant (Table 3).

Separated analyses in Sierra Nevada showed a negative relationship of *Leucocytozoon* prevalence with latitude and a slight positive relationship with longitude. Contrary to Picos de Europa, our results in Sierra Nevada provided a negative association with temperature variables. Moreover, there was a general trend to increase *Leucocytozoon* prevalence and richness in the proximity of areas with water irrespective of its origin. *Leucocytozoon* prevalence and richness decreased with most rainfall variables. Finally, terrain steepness was a positive predictor of prevalence and richness in both mountain ranges.

## Discussion

In this study we aimed to unravel the biotic and abiotic features determining prevalence and richness in avian haemosporidians in both Mediterranean and Atlantic temperate mountain ranges. As expected, we confirmed the importance of climatic variables explaining prevalence and richness on Iberian mountains. However, landscape variables and other factors, namely host richness and migration, explained even more variation than climatic factors (Fig 3). Our results enrich previous findings recorded for blackcaps in the Iberian Peninsula, where the inclusion of predictors beyond the climatic variables improved the understanding of avian haemosporidian distribution [24]. Interestingly, we found significant differences with the effect direction of some variables within haemosporidians and between mountain ranges. Such findings suggest that local climatic conditions, especially landscape features, are dissimilarly driving transmission and persistence of avian haemosporidians on each mountain range. Reasons explaining such environmental specialisation could be related to the invertebrate host specialisation and/or by haemosporidian life-history characteristics [23]. Remarkably, our findings revealed some differences in relation to previous studies evaluating single species or singular avian communities [1, 24–26, 57]. Thus, the presence of *Plasmodium* had been mostly explained with few predictors, namely the temperature of the warmest month [1, 25–26] and rainfall of the warmest months [24]. Our *Plasmodium* results display a richer mosaic with a landscape characterised of open and low-altitude humanised habitats. In addition, we found higher *Plasmodium* prevalence and richness nearby water sources in Sierra Nevada, but an opposite pattern in Picos de Europa. This discrepancy may be due to: (1) the fact that water availability could be a limiting factor for vector larval development only on the dry Mediterranean mountain range (i.e. Sierra Nevada), but irrelevant in the humid Atlantic mountains (i.e. Picos de Europa) [58–59]; and (2) temperature values around Picos de Europa water sources are below the optimal needed for *Plasmodium* to complete their life cycles [25]. Anthropogenic land use (i.e. agricultural and urban areas) showed a positive influence on *Plasmodium* in Sierra Nevada but it was inconsequential in Picos de Europa. This result may again be explained in light of water availability, which is expected to be higher in cultivated lands where irrigation is practiced. Alternatively, the type and intensity of crops managed around both mountain ranges could be behind such a result [60]. Interestingly, *Plasmodium* richness and prevalence decreased with the avian host richness in both mountain ranges, suggesting that *Plasmodium* is somehow being influenced by the specific characteristics of localities, host composition and/or vector ecology instead of avian host richness [26, 61, 62].

*Haemoproteus* was found to thrive in woodlands on both mountain ranges, which contrasts with the preferences for open habitats selected for *Plasmodium*. In Sierra Nevada *Haemoproteus* increased with rainfall variables and decreased with temperature, however, we found the opposite pattern in Picos de Europa. These findings are likely to reflect some adaptation of avian haemosporidian parasites and their dipteran vectors to the climatic particularities of both mountains [24–25]. Given the very diverse climate conditions occurring on both mountain massifs, our findings could reflect that *Haemoproteus* is being favoured by moderate values of temperature and rainfall [24].

*Leucocytozoon* was the only haemosporidian genus showing significant differences between both mountain ranges. Sierra Nevada reached the highest prevalence and richness values (Fig 3). Such a result reveals that the Mediterranean mountain range provides more favourable environmental (biotic and abiotic) conditions for local amplification and transmission of this parasite than Picos de Europa. Similar to the *Haemoproteus* results, *Leucocytozoon* was found to thrive in forests throughout the whole study area. In Picos de Europa, but not in Sierra Nevada, *Leucocytozoon* was found to increase with temperature and decrease with rainfall. These results seem to reflect that the whole Sierra Nevada, but only the warmest and driest areas of Picos de Europa are the most favourable areas for *Leucocytozoon*. This pattern contrasts with recent studies where *Leucocytozoon* prevalence was related to higher altitudes [24, 32]. Such a discrepancy is probably a reflection of the different ecological habitats (chiefly woodlands) studied by those authors, and the ecological constrains of *Leucocytozoon*, which is transmitted by blackflies [14]. In the present study we analysed avian communities inhabiting both open and woodland habitats across an altitudinal gradient, which represented a wider ecological spectrum than previous studies [24, 26]. Indeed, our results are in agreement with expected black fly distribution over wide altitudinal gradients [63].

Regarding avian host community, *Leucocytozoon* showed a significant relationship with the number of bird species sampled, supporting the idea of the different environmental specialisation of each haemosporidian genus [24]. *Leucocytozoon* and *Haemoproteus* (only in Picos de Europa) showed a negative association with host migratory behaviour, thriving with the presence of sedentary species. This result could be due to three non-exclusive explanations. Firstly, *Leucocytozoon* and *Haemoproteus* parasites experience a heterogeneous distribution across Sub-Saharan regions, which could make the wintering transmission of these haemosporidians on the migratory birds difficult [64]. Secondly, vectors transmitting these parasites on wintering grounds occur on different habitats in their breeding grounds. Thirdly, there is a specific association between the dipteran vectors and haemosporidian lineages making the transmission of the African lineages within the Iberian Peninsula difficult [9]. Slopes were consistently associated with higher prevalence and richness values for *Leucocytozoon* in both mountain ranges. This finding seems to be related to the habitat requirements of blackflies, which need unpolluted streams and rivers for achieving an optimal larvae development [65].

Overall, our results confirm that the distribution of avian haemosporidian community in the temperate mountains studied here is far from homogenous. Each genus of these blood parasites showed different environmental preferences, which must be interpreted as a consequence of the distribution and ecological preferences of their avian and dipteran hosts. Our findings confirm that host-parasite associations are complex systems, and results achieved with single species or with restricted subsets of avian host species cannot be generalised to other host-parasite systems [49]. Models forecasting avian haemosporidian distribution, and hence predicting changes in bird distribution, have recently burgeoned [2, 37, 66]. In general, these models foresee changes in haemosporidian distribution and prevalence associated with increasing global temperatures [5]. Indeed, such predictions are especially dramatic on oceanic islands due to the high number of endemic taxa that could be affected. For instance, the prediction for Hawaii is an uphill shift in vector elevational distribution responding to an increase in temperature and rainfall. Such a climatic forecast provides a pessimistic scenario with significant decreases in abundance and diversity of endemic avian communities over the future years [37]. These expectations are only based on climatic models because other key factors (e.g. anthropogenic) which are also known to shape avian and dipteran host distributions [24–26] seem difficult to include in the haemosporidian models. However, the exclusion of these key variables could overestimate the effect of climate change on the transmission of the haemosporidians. For instance, it is noticeable that the prevalence levels of *Plasmodium* found on both mountain ranges is low (< 10%), especially on Sierra Nevada which is the southern mountain range (Fig 3). Based on most of the aforementioned *Plasmodium* models we would expect to find higher prevalence values according with the level of global warming (2). In fact, a significant increase of temperature at low (1.2°C) and middle (1.3°C) elevations has been reported in Sierra Nevada over a 14 year period (1992-1993/2007), and to a lesser extent (0.9°C) in Picos de Europa. Such significant change has produced an uphill shift in the dung beetle and bumblebee communities in Sierra Nevada and Picos de Europa, respectively [59, 67]. A similar change in vector distribution could be expected in Sierra Nevada and Picos de Europa [36]. Nevertheless, according to the low values obtained in this study (there is not a prevalence information before 2012–2013) we can speculate that *Plasmodium* prevalence levels do not seem to track increasing temperature in Sierra Nevada and Picos de Europa. This result is striking but highlights the complexity of understanding the haemosporidian biology where two different hosts (vertebrate and diptera) are needed to complete the life cycle of parasite, and where all of them can be affected by environmental changes driven by human activity. Basic research on factors determining haemosporidian distribution and prevalence on wider heterogeneous gradients at the community level are now needed, along with experiments to understand the environmental, genetic, and epigenetic factors limiting the parasite development on their dipteran and avian hosts.

## Supporting information

S1 Table Basic information on localities studied. Geographical information, habitat type, annual mean temperature (BIO1), annual precipitation (BIO12), and altitude of each locality studied on Sierra Nevada and Picos de Europa. N: number of birds analysed.
(DOCX)

S2 Table Sierra Nevada PLS results. Partial Least Squares regression results for *Plasmodium*, *Haemoproteus and Leucocytozoon* prevalence and richness analyses performed for Sierra Nevada. Weights of predictors (independent variables) are related to prevalence and richness (dependent variables) for each parasite genera. Values found to contribute significantly to the component obtained are shown in bold. ^**§**^: Significant component.
(DOCX)

S3 Table Picos de Europa PLS results. Partial Least Squares regression results for *Plasmodium*, *Haemoproteus and Leucocytozoon* prevalence and richness analyses performed for Picos de Europa. Weights of predictors (independent variables) are related to prevalence and richness (dependent variables) for each parasite genera. Values found to contribute significantly to the component obtained are shown in bold. ^**§**^: Significant component.
(DOCX)

S4 Table Combined PLS results. Partial Least Squares regression results for *Plasmodium*, *Haemoproteus and Leucocytozoon* prevalence and richness analyses performed with the pooled analysis (Sierra Nevada and Picos de Europa). Weights of predictors (independent variables) are related to prevalence and richness (dependent variables) for each parasite genera. Values found to contribute significantly to the component obtained are shown in bold. ^**§**^: Significant component.
(DOCX)
