# Supplementary material for: Factors governing the prevalence and richness of avian haemosporidian communities within and between temperate mountains
Source: PLoS One. 2017 Sep 7;12(9):e0184587. doi: 10.1371/journal.pone.0184587 (PMC5589241; doi:10.1371/journal.pone.0184587)
Supplement: S1 Table — Geographical information, habitat type, annual mean temperature (BIO1), annual precipitation (BIO12), and altitude of each locality studied on Sierra Nevada and Picos de Europa. N: number of birds analysed. (DOCX) [file pone.0184587.s001.docx]

**S1 Table**.

| **Localities** | **Latitude** | **Longitude** | **Habitat** | **BIO1 (°C)** | **BIO12 (mm)** | **Altitude (m)** | **N** |
| --- | --- | --- | --- | --- | --- | --- | --- |
| **SIERRA NEVADA** |  |  |  |  |  |  |  |
| Maro Forest | 425920 | 4068002 | Forest | 17 | 380 | 107 | 25 |
| Aguacates | 427787 | 4068732 | Forest | 14.4 | 379 | 60 | 26 |
| Almuñecar | 431960 | 4067052 | Forest | 17.5 | 368 | 199 | 34 |
| Maro | 426284 | 4068481 | Open | 17.5 | 380 | 149 | 24 |
| Maro2 | 427110 | 4068050 | Open | 17.4 | 394 | 58 | 17 |
| Miel | 429038 | 4070983 | Open | 16.8 | 393 | 356 | 19 |
| Menchón | 467309 | 4088005 | Forest | 13.4 | 526 | 966 | 23 |
| Pinos Genil | 453607 | 4112972 | Forest | 14,9 | 478 | 760 | 26 |
| Guadix | 489337 | 4126207 | Forest | 14.1 | 482 | 961 | 18 |
| Lanjarón | 459518 | 4085812 | Open | 13.6 | 515 | 885 | 21 |
| Cáñar | 462557 | 4087651 | Open | 12.1 | 600 | 1170 | 26 |
| Padul | 439938 | 4098923 | Open | 14.0 | 527 | 954 | 40 |
| Carneros | 477963 | 4116255 | Forest | 10.9 | 662 | 1423 | 24 |
| Busquistar | 475199 | 4089650 | Forest | 12.5 | 566 | 1340 | 24 |
| Jerez | 485470 | 4114802 | Forest | 12.6 | 558 | 1250 | 33 |
| Monachil1 | 458215 | 4110007 | Open | 11.6 | 646 | 1494 | 20 |
| Monachil2 | 457183 | 4110116 | Open | 11.8 | 635 | 1394 | 19 |
| Luna | 494182 | 4111971 | Open | 11.5 | 610 | 1388 | 24 |
| Cañadillas | 462527 | 4109009 | Forest | 9.5 | 762 | 1911 | 28 |
| El Robledal | 462465 | 4091060 | Forest | 9.6 | 751 | 1835 | 41 |
| La Ragua | 497399 | 4107677 | Forest | 8.4 | 796 | 2048 | 63 |
| Cáñar2 | 461742 | 4091165 | Open | 9.0 | 789 | 1952 | 19 |
| Mirador | 461680 | 4108656 | Open | 8.1 | 845 | 2090 | 19 |
| Camarate | 476682 | 4114011 | Open | 9.2 | 765 | 1970 | 30 |
| **PICOS DE EUROPA** |  |  |  |  |  |  |  |
| Pría | 341059 | 4813668 | Open | 14.0 | 818 | 41 | 24 |
| Luanco | 274309 | 4834599 | Open | 13.8 | 855 | 28 | 25 |
| Cabo Peñas | 269927 | 4837140 | Open | 13.5 | 878 | 116 | 19 |
| Llames | 340510 | 4812763 | Forest | 14.1 | 814 | 42 | 19 |
| Covadonga | 333671 | 4796854 | Forest | 12.2 | 823 | 269 | 21 |
| Mirador del Fito | 322509 | 4812108 | Forest | 11.5 | 890 | 545 | 18 |
| Llano Enol | 338132 | 4792634 | Open | 8.8 | 933 | 1070 | 86 |
| Vega de Sotres | 360274 | 4788672 | Open | 7.8 | 984 | 1094 | 37 |
| Belbín | 340852 | 4793022 | Open | 9.0 | 925 | 1082 | 72 |
| Collado Pandébano | 355476 | 4788116 | Forest | 8.6 | 939 | 1110 | 31 |
| Sotres | 357437 | 4787821 | Forest | 9.8 | 891 | 857 | 30 |
| Bosque Enol | 337914 | 4792228 | Forest | 8.9 | 927 | 1074 | 22 |
| Tresviso | 360461 | 4788606 | Open | 7.8 | 984 | 1265 | 70 |
| Vega Llós | 340974 | 4781052 | Open | 6.0 | 1057 | 1570 | 32 |
| Puertos de Áliva | 354991 | 4780903 | Open | 6.3 | 1040 | 1561 | 60 |
| Pinar de Lillo | 317186 | 4771572 | Forest | 6.3 | 1009 | 1620 | 27 |
| Pandetrave_km_13 | 347911 | 4776347 | Forest | 6.9 | 983 | 1485 | 20 |
| Puerto del Pontón | 335725 | 4773947 | Forest | 8.9 | 864 | 1292 | 16 |
| Vega Ario | 344054 | 4789759 | Open | 6.1 | 1076 | 1600 | 37 |
| Andara | 361051 | 4785642 | Open | 5.2 | 1132 | 1900 | 46 |
| Urriellu | 351959 | 4784910 | Open | 4.7 | 1164 | 2040 | 31 |
| Vega Huerta | 340799 | 4784238 | Open | 4.1 | 1205 | 2000 | 59 |
