# Supplementary material for: Factors governing the prevalence and richness of avian haemosporidian communities within and between temperate mountains
Source: PLoS One. 2017 Sep 7;12(9):e0184587. doi: 10.1371/journal.pone.0184587 (PMC5589241; doi:10.1371/journal.pone.0184587)
Supplement: S3 Table — Partial Least Squares regression results for Plasmodium, Haemoproteus and Leucocytozoon prevalence and richness analyses performed for Picos de Europa. Weights of predictors (independent variables) are related to prevalence and richness (dependent variables) for each parasite genera. Values found to contribute significantly to the component obtained are shown in bold. §: Significant component. (DOCX) [file pone.0184587.s003.docx]

**S3** **Table**.

| **Predictors** | ***Plasmodium*** | | | | ***Haemoproteus*** | |
| --- | --- | --- | --- | --- | --- | --- |
|  | **Prevalence** | | **Richness** | | **Prevalence** | **Richness** |
|  | 1st Component**^§^** | 2nd Component**^§^** | 1st Component**^§^** | 2nd Component**^§^** |  |  |
| **Confounding variable** |  |  |  |  |  |  |
| Sex ratio | -0.102 | -0.122 | -0.123 | -0.121 | -0.020 | 0.047 |
| **Geographical features** |  |  |  |  |  |  |
| Longitude (X) | **-0.187** | -0.095 | **-0.181** | -0.023 | 0.043 | 0.109 |
| Latitude (Y) | **0.208** | 0.091 | **0.223** | 0.070 | -0.089 | -0.130 |
| X*Y | **-0.180** | -0.081 | **-0.171** | -0.006 | 0.030 | 0.091 |
| X^2^ | **-0.187** | -0.103 | **-0.181** | -0.026 | 0.049 | 0.112 |
| Y^2^ | **0.188** | **0.156** | **0.197** | 0.098 | -0.083 | -0.135 |
| X^2^*Y | **-0.183** | -0.100 | **-0.178** | -0.027 | 0.047 | 0.112 |
| X*Y^2^ | **-0.179** | -0.103 | **-0.175** | -0.036 | 0.028 | 0.094 |
| X^3^ | **-0.186** | -0.095 | **-0.179** | -0.020 | 0.043 | 0.107 |
| Y^3^ | **0.207** | 0.101 | **0.217** | 0.065 | -0.070 | -0.112 |
| **Climatic** |  |  |  |  |  |  |
| ***Temperature*** |  |  |  |  |  |  |
| Annual mean | **0.146** | **-0.147** | 0.136 | -0.131 | 0.136 | 0.11 |
| Mean diurnal range | **-0.182** | 0.014 | **-0.196** | -0.003 | 0.045 | 0.071 |
| Isothermality | **0.184** | 0.028 | **0.175** | -0.019 | 0.096 | 0.039 |
| Temperature seasonality | **-0.200** | -0.003 | **-0.207** | 0.002 | 0.017 | 0.050 |
| Max. temperature warmest month | 0.111 | **-0.209** | 0.093 | **-0.186** | **0.203** | **0.186** |
| Min. temperature coldest month | 0.064 | 0.106 | 0.044 | -0.009 | **-0.248** | **-0.294** |
| Annual Range | **-0.195** | 0.002 | **-0.205** | -0.001 | 0.032 | 0.063 |
| Mean temperature wettest quarter | 0.137 | **-0.151** | 0.125 | -0.138 | **0.145** | 0.132 |
| Mean temperature driest quarter | **0.144** | **-0.158** | 0.134 | -0.139 | 0.135 | 0.114 |
| Mean temperature warmest quarter (bio10) | **0.145** | **-0.156** | 0.136 | -0.134 | 0.133 | 0.114 |
| Mean temperature coldest quarter | 0.124 | -0.111 | 0.120 | -0.091 | 0.120 | 0.103 |
| ***Precipitation*** |  |  |  |  |  |  |
| Annual precipitation | -0.103 | **0.231** | -0.084 | **0.204** | **-0.212** | **-0.197** |
| Precipitation of wettest month | -0.030 | **0.303** | -0.001 | **0.263** | **-0.286** | **-0.287** |
| Precipitation of driest month | -0.124 | **0.209** | -0.110 | **0.180** | **-0.166** | **-0.150** |
| Precipitation seasonality | **0.164** | -0.118 | **0.158** | -0.106 | 0.087 | 0.051 |
| Precipitation of wettest quarter | -0.096 | **0.232** | -0.074 | **0.210** | **-0.229** | **-0.214** |
| Precipitation of driest quarter | -0.131 | **0.196** | -0.117 | **0.172** | **-0.163** | -0.141 |
| Precipitation of warmest quarter | -0.107 | **0.231** | -0.086 | **0.208** | **-0.210** | **-0.195** |
| Precipitation of coldest quarter | -0.115 | **0.207** | -0.097 | **0.186** | **-0.196** | **-0.181** |
| **Landscape features** |  |  |  |  |  |  |
| Altitude | **-0.168** | 0.091 | **-0.174** | 0.066 | -0.007 | 0.027 |
| North-South Orientation | -0.054 | 0.044 | 0.002 | **0.188** | -0.055 | 0.007 |
| East-West Orientation | -0.036 | **-0.165** | -0.064 | **-0.176** | 0.119 | 0.071 |
| Slope | **-0.163** | -0.006 | **-0.189** | -0.052 | 0.005 | 0.062 |
| Distance to permanent rivers | 0.095 | **0.155** | 0.090 | 0.063 | **-0.338** | **-0.249** |
| Distance to temporary rivers | 0.019 | 0.098 | 0.065 | **0.178** | **-0.208** | **-0.214** |
| Distance to water channels | 0.006 | -0.010 | -0.012 | -0.046 | 0.047 | 0.093 |
| Distance to permanent lakes | 0.096 | **0.171** | 0.094 | 0.092 | -0.083 | -0.071 |
| Distance to temporary lakes | 0.094 | -0.012 | **0.151** | 0.136 | 0.022 | -0.013 |
| Distance to reservoir waters | **0.197** | 0.069 | **0.189** | -0.006 | -0.058 | -0.120 |
| Distance to drinking fountains | **0.171** | 0.033 | **0.145** | -0.071 | -0.007 | -0.010 |
| Distance to spring water | **0.171** | 0.033 | **0.145** | -0.071 | -0.007 | -0.010 |
| Distance to water tanks | **0.159** | -0.031 | 0.139 | -0.091 | 0.006 | -0.058 |
| Distance to water wells | **0.167** | **0.171** | **0.156** | 0.057 | -0.136 | -0.086 |
| Distance to ponds | -0.123 | -0.134 | **-0.178** | **-0.218** | **0.142** | **0.200** |
| Distance to urban areas | -0.141 | 0.001 | -0.128 | 0.059 | 0.033 | 0.046 |
| Percentage of agricultural areas | 0.060 | -0.123 | 0.059 | -0.091 | -0.022 | -0.028 |
| Percentage of forests | -0.051 | -0.104 | -0.119 | **-0.234** | **0.238** | **0.189** |
| Percentage of soil | -0.053 | 0.084 | -0.028 | 0.115 | **-0.150** | **-0.159** |
| Percentage of shrubs | 0.076 | **0.188** | 0.099 | **0.180** | -0.139 | -0.019 |
| **Behaviour** |  |  |  |  |  |  |
| Migration | -0.053 | **0.207** | 0.054 | **0.423** | **-0.269** | **-0.206** |
| **Number of potential hosts** |  |  |  |  |  |  |
| Host richness | -0.075 | **-0.247** | -0.094 | **-0.190** | **0.191** | **0.303** |

| **Predictors** | ***Leucocytozoon*** | | | | |
| --- | --- | --- | --- | --- | --- |
|  | **Prevalence** | | | **Richness** | |
|  | 1st Component**^§^** | 2nd Component**^§^** | 3rd Component | 1st Component**^§^** | 2nd Component**^§^** |
| **Confounding variable** |  |  |  |  |  |
| Sex ratio | 0.000 | 0.114 | 0.024 | -0.027 | 0.079 |
| **Geographical features** |  |  |  |  |  |
| Longitude (X) | -0.049 | 0.133 | **-0.182** | -0.027 | **0.157** |
| Latitude (Y) | 0.039 | **-0.212** | 0.074 | 0.029 | **-0.226** |
| X*Y | -0.068 | 0.105 | **-0.201** | -0.045 | 0.131 |
| X^2^ | -0.047 | 0.133 | **-0.188** | -0.024 | **0.157** |
| Y^2^ | -0.063 | **-0.276** | -0.016 | -0.043 | **-0.235** |
| X^2^*Y | -0.045 | 0.129 | **-0.187** | -0.022 | **0.154** |
| X*Y^2^ | -0.059 | 0.104 | **-0.203** | -0.032 | 0.136 |
| X^3^ | -0.045 | 0.137 | **-0.174** | -0.025 | **0.157** |
| Y^3^ | 0.129 | -0.073 | **0.270** | 0.070 | **-0.160** |
| **Climatic** |  |  |  |  |  |
| ***Temperature*** |  |  |  |  |  |
| Annual mean | **0.186** | -0.085 | -0.090 | **0.194** | -0.078 |
| Mean diurnal range | -0.055 | **0.219** | 0.044 | -0.068 | **0.196** |
| Isothermality | **0.178** | -0.022 | **0.205** | **0.156** | -0.055 |
| Temperature seasonality | -0.096 | **0.182** | -0.011 | -0.096 | **0.181** |
| Max. temperature warmest month | **0.223** | -0.022 | -0.113 | **0.232** | -0.016 |
| Min. temperature coldest month | -0.083 | -0.094 | **0.200** | **-0.147** | **-0.178** |
| Annual Range | -0.077 | **0.201** | 0.008 | -0.082 | **0.191** |
| Mean temperature wettest quarter | **0.185** | -0.081 | -0.112 | **0.194** | -0.073 |
| Mean temperature driest quarter | **0.186** | -0.086 | -0.090 | **0.195** | -0.079 |
| Mean temperature warmest quarter | **0.183** | -0.091 | -0.096 | **0.194** | -0.079 |
| Mean temperature coldest quarter | **0.155** | -0.061 | -0.012 | **0.166** | -0.049 |
| ***Precipitation*** |  |  |  |  |  |
| Annual precipitation | **-0.222** | 0.024 | 0.131 | **-0.236** | 0.009 |
| Precipitation of wettest month | **-0.227** | -0.040 | **0.230** | **-0.253** | -0.070 |
| Precipitation of driest month | **-0.197** | 0.069 | 0.124 | **-0.213** | 0.048 |
| Precipitation seasonality | **0.182** | -0.077 | 0.035 | **0.183** | -0.073 |
| Precipitation of wettest quarter | **-0.222** | 0.018 | **0.151** | **-0.238** | 0.001 |
| Precipitation of driest quarter | **-0.197** | 0.072 | 0.110 | **-0.212** | 0.051 |
| Precipitation of warmest quarter | **-0.212** | 0.041 | **0.145** | **-0.231** | 0.015 |
| Precipitation of coldest quarter | **-0.222** | 0.027 | 0.103 | **-0.231** | 0.020 |
| **Landscape features** |  |  |  |  |  |
| Altitude | -0.066 | **0.222** | 0.097 | -0.094 | **0.176** |
| North-South Orientation | -0.060 | 0.021 | -0.075 | 0.001 | 0.121 |
| East-West Orientation | 0.033 | 0.001 | -0.115 | 0.020 | -0.022 |
| Slope | 0.021 | **0.279** | 0.117 | -0.011 | **0.222** |
| Distance to permanent rivers | -0.073 | **-0.149** | 0.053 | -0.082 | **-0.150** |
| Distance to temporary rivers | -0.111 | -0.099 | 0.075 | **-0.176** | **-0.199** |
| Distance to water channels | 0.103 | 0.127 | 0.108 | 0.044 | 0.043 |
| Distance to permanent lakes | 0.109 | 0.077 | **0.217** | -0.005 | -0.099 |
| Distance to temporary lakes | 0.072 | -0.101 | **-0.165** | 0.068 | -0.112 |
| Distance to reservoir waters | 0.097 | -0.102 | **0.268** | 0.069 | -0.143 |
| Distance to drinking fountains | 0.026 | **-0.185** | 0.078 | 0.019 | **-0.188** |
| Distance to spring water | 0.026 | **-0.185** | 0.078 | 0.019 | **-0.188** |
| Distance to water tanks | 0.125 | -0.075 | 0.126 | 0.094 | -0.118 |
| Distance to water wells | 0.002 | **-0.155** | 0.057 | -0.033 | **-0.197** |
| Distance to ponds | -0.018 | 0.100 | -0.061 | 0.006 | **0.134** |
| Distance to urban areas | -0.063 | 0.108 | -0.140 | -0.087 | 0.071 |
| Percentage of agricultural areas | 0.066 | -0.087 | -0.094 | 0.086 | -0.056 |
| Percentage of forests | **0.253** | **0.360** | **0.238** | **0.194** | **0.275** |
| Percentage of soil | **-0.239** | **-0.177** | -0.118 | **-0.175** | -0.087 |
| Percentage of shrubs | -0.000 | -0.025 | 0.063 | -0.071 | -0.131 |
| **Behaviour** |  |  |  |  |  |
| Migration | **-0.283** | **-0.200** | **-0.232** | **-0.249** | **-0.145** |
| **Number of potential hosts** |  |  |  |  |  |
| Host richness | **0.203** | **0.212** | -0.101 | **0.234** | **0.255** |
